# Comparative Transcriptome and Weighted Gene Co-Expression Network Analyses Reveal a Shared Core Response and Quantitatively Divergent Regulatory Modules Between Drought-Tolerant and Drought-Sensitive Foxtail Millet

**DOI:** 10.3390/biology15191757

**Published:** 2026-10-02

**Authors:** Hong-Jin Wang, Yun Zhao, Zaituniguli Kuerban, Hui Wang, Xiangwei Hu, Guojun Feng

**Affiliations:** 1 Institute of Crop Research, Academy of Agricultural Sciences of Xinjiang Uyghur Autonomous Region, Urumqi 830002, China; wanghongjin@nwafu.edu.cn (H.-J.W.); zhaoyun@xaas.ac.cn (Y.Z.); ztngl@xaas.ac.cn (Z.K.); wanghui@xaas.ac.cn (H.W.); 2 State Key Laboratory for Crop Stress Resistance and High-Efficiency Production, College of Life Sciences, Northwest Agriculture and Forestry University, Yangling, Xianyang 712100, China

**Keywords:** foxtail millet, drought stress, comparative transcriptomics, differential expression, WGCNA, co-expression modules, core genes

## Abstract

Drought limits the stability of foxtail millet production and the efficient use of germplasm resources, yet whether drought-tolerant and drought-sensitive accessions engage fundamentally distinct pathways or a shared, quantitatively reprogrammed network remains unclear. We generated 36 leaf transcriptomes from three drought-sensitive (DSEN) and three drought-tolerant (DTOL) accessions under well-watered (WW) and drought-stress (DS) conditions, retaining 25,099 genes for downstream analyses. Principal component analysis confirmed water treatment as the primary driver of global expression variation. DS-versus-WW comparisons identified 10,635 and 6215 drought-responsive DEGs in the DSEN and DTOL groups, respectively, with 5195 shared and a genome-wide response correlation of 0.791. Crucially, accession-level tests revealed no significant DEGs between groups under WW, only 22 under DS, and none for the group × drought-response interaction, underscoring the absence of qualitative pathway shifts. Functional enrichment analyses jointly indicated suppression of photosynthesis and translation, accompanied by reprogramming of carbohydrate metabolism, membrane lipid remodeling, phenylpropanoid and secondary metabolism, protein homeostasis, and stress signaling. WGCNA partitioned 10,000 highly variable genes into 16 modules. The blue module, which showed the strongest drought response in DSEN, was enriched for cell-wall and carbohydrate metabolism, membrane lipid processes, and stress-protection pathways, whereas the brown module, which responded most robustly in DTOL, was enriched for calcium signaling, protein phosphorylation/dephosphorylation, transcriptional regulation, proline biosynthesis, and dehydration protection. The yellow and cyan modules exhibited the largest between-group effect sizes under drought but failed multiple-testing correction, remaining candidate networks. Collectively, our results support a model of shared core response with divergent regulatory intensity, where sensitive accessions undergo broader transcriptional perturbation, while tolerant ones deploy more concentrated and coordinated modules. This study provides modular targets for drought-tolerance breeding and highlights the necessity of accession-level replication and conservative statistical inference in comparative transcriptomics.

## Simple Summary

Foxtail millet is a small grain crop that grows well in dry regions, making it an important food and animal feed source in areas where water is scarce. However, not all varieties of this crop handle drought equally well, and the reasons for these differences are not fully understood. In this study, we grew three drought-sensitive and three drought-tolerant lines of foxtail millet and compared their leaf activity under normal watering and under two weeks without water. By examining the activity of thousands of genes at once, we found that both groups of plants responded to drought in largely the same way, by reducing their investment in photosynthesis and adjusting how they manage energy, cell walls, and stress protection. The main difference was not in which responses occurred but in how strongly and broadly they occurred. The sensitive plants showed larger and more widespread changes, while the tolerant plants used a smaller, more tightly coordinated set of responses, particularly involving calcium signals, hormone regulation, and protective molecules. These findings help explain what makes some millet varieties better at surviving drought and point to specific genes that plant breeders could target to develop more drought-resistant crops in the future.

## 1. Introduction

Foxtail millet (*Setaria italica* L.) has a relatively short growth duration, high water-use efficiency and strong adaptation to dryland environments, making it an important food and forage crop in arid and semi-arid regions [1,2,3] and a model species for studying stress adaptation in C4 grasses [4,5,6]. Against the backdrop of global climate change, uneven spatiotemporal distribution of precipitation and an increasing frequency of periodic drought pose continuing risks to seedling establishment, photosynthetic carbon assimilation and yield formation [7,8,9,10]. Elucidating the molecular basis of drought tolerance in foxtail millet and establishing gene and network resources applicable to breeding selection is therefore of both fundamental and applied importance [11,12].

Plant responses to drought involve multiple layers of processes, including stomatal regulation, osmotic protection, reactive oxygen species homeostasis, membrane lipid and cell-wall remodeling, protein homeostasis, and abscisic acid, ethylene, and calcium signaling [13,14,15]. In foxtail millet, genes such as *SiNCED1* [16], *SiCDPK24* [17], the NF-Y family [18,19], the *SNARE* family [20], and *SiMBR2* [21] have been shown or suggested to participate in ABA biosynthesis, calcium-dependent signaling, vesicle trafficking, transcriptional regulation, and protein homeostasis. Recent transcriptomic studies have also revealed marked differences among foxtail millet genotypes in photosynthesis, phenylpropanoid metabolism, carbohydrate metabolism, and antioxidant systems [22,23,24,25]. However, comparing DS with WW conditions within a single genotype tends to conflate a general stress response with genuine differences in drought tolerance; furthermore, treating sequencing replicates directly as independent between-group replicates can inflate statistical significance [26,27]. Therefore, when comparing accessions differing in drought tolerance, it is necessary to simultaneously control for accession effects, distinguish technical or biological replicates from independent genetic materials, and separately model the shared response and group-level differences.

Differential expression analysis can identify genes affected by treatment but cannot directly reveal coordinated changes among genes. Weighted gene co-expression network analysis (WGCNA) summarizes large-scale expression changes into modules and can identify co-expression modules and highly connected genes associated with a trait of interest [28,29]; it has been applied to drought-response studies in buckwheat [30], sweet potato [31], maize [32], and sorghum [33]. The advantage of network analysis lies in its ability to extract coordinated regulatory structure from continuous expression variation, making it particularly suited to differences between accessions that share a common direction of change but differ in response magnitude. Nevertheless, module–trait correlations remain associative evidence; reliable candidate mechanisms can only be established by combining them with rigorous between-group statistics, functional enrichment, and independent validation [28,34].

In this study, three relatively drought-sensitive and three relatively drought-tolerant cultivated foxtail millet breeding lines from Xinjiang were subjected to WW and DS treatments, generating 36 leaf transcriptomes. The objectives were: first, to compare the scale, direction, and degree of sharing of drought responses between the two groups; second, to characterize functional changes in photosynthesis, carbon metabolism, membrane systems, and signaling regulation; third, to use WGCNA to identify key modules and core genes associated with the responses of the DSEN and DTOL groups; and fourth, to evaluate, within an accession-level inference framework, whether the two groups exhibit rigorously significant, group-specific responses. Through the combined analysis of differential expression, functional enrichment, and co-expression networks, this study proposes that drought-tolerance differences in foxtail millet are better described as quantitative reprogramming of a shared core network rather than as two entirely distinct binary regulatory systems.

## 2. Materials and Methods

### 2.1. Plant Materials and Drought Treatment

All plant materials used were cultivated foxtail millet (*Setaria italica* L.) P. Beauv, independently bred and stably maintained by our team from local Xinjiang foxtail millet germplasm. The identity of the materials was confirmed by our foxtail millet breeding and germplasm research staff based on breeding pedigrees, seed records and major morphological characteristics; seeds were preserved and provided by the Institute of Crop Sciences, Xinjiang Academy of Agricultural Sciences (Urumqi, China). All materials were cultivated types, and no wild-plant collection or wild-resource collection permits were involved. Based on multi-year drought-tolerance evaluation data accumulated previously, three relatively drought-sensitive accessions (numbered DSEN-1, DSEN-2, and DSEN-3) and three relatively drought-tolerant accessions (numbered DTOL-1, DTOL-2, and DTOL-3) were selected [3,10].

All materials were grown under controlled conditions in a growth chamber at the Institute of Crop Research, Academy of Agricultural Sciences of Xinjiang Uyghur Autonomous Region (Urumqi, Xinjiang Uyghur Autonomous Region, China), with day/night temperatures of 25 ± 2 °C and 20 ± 2 °C, a photoperiod of 16 h light/8 h dark, and relative humidity of approximately 60%. At the three-leaf stage, plants were subjected to two treatments: well-watered (WW) and drought stress (DS). The WW treatment maintained soil volumetric water content at 60–70% of field capacity through regular irrigation; the DS treatment involved complete withholding of water for 2 weeks, allowing soil volumetric water content to gradually decline to 15–20% of field capacity, monitored with soil moisture sensors. At the end of the treatment period, the 2nd–3rd fully expanded functional leaves from the top of each plant were collected. Each biological replicate consisted of an equal-amount pooled sample of leaves from 5 to 8 plants with uniform growth; samples were immediately snap-frozen in liquid nitrogen and stored at −80 °C. Three independent biological replicates were set for each accession under each treatment, yielding 6 accessions × 2 treatments × 3 replicates = 36 transcriptome samples in total. This experimental design is consistent with our team’s previously published foxtail millet drought-tolerance study [35].

### 2.2. RNA Extraction, Library Construction, and Sequencing

Total RNA extraction, quality assessment, library construction, and high-throughput sequencing from leaf samples were performed by the same commercial sequencing service provider used in our team’s previous studies [35]. RNA was extracted and libraries were constructed independently for each biological replicate, without pooling different replicates. After total RNA passed concentration, purity, and integrity assessments, mRNA was enriched using oligo(dT) magnetic beads, followed sequentially by mRNA fragmentation, first- and second-strand cDNA synthesis, end repair, A-tailing, adapter ligation and PCR amplification. Qualified libraries were subjected to paired-end high-throughput sequencing.

### 2.3. Raw Data Quality Control, Genome Alignment, and Expression Quantification

Raw FASTQ data were filtered using fastp v0.23.2, removing reads containing more than 5 N bases, reads with more than 40% low-quality bases (Q < 15), reads with adapter contamination, or reads shorter than 30 bp [36]. FastQC v0.11.9 was used to assess base quality, GC content, and sequence quality distribution. Clean reads were aligned to the foxtail millet reference genome *Setaria italica* v2.2 using STAR v2.7.10a [4,37], and total alignment rate, unique mapping rate, and unmapped rate were calculated. Transcript assembly was performed with StringTie v2.2.1, and merged transcripts were compared with the reference annotation using gffcompare v0.12.6; coding regions of potential novel transcripts were predicted with TransDecoder v5.5.0 [38]. Gene- and transcript-level expression was quantified using RSEM v1.3.3 [39]. FPKM values were used for visualization of expression patterns, and raw read counts were used for statistical testing.

### 2.4. Expression Data Filtering and Overall Sample Structure Analysis

All statistical analyses were performed in R v4.6.1. A DGEList object was constructed using edgeR v4.10.1, with the “accession × treatment” combination as the grouping factor; lowly expressed genes were removed using filterByExpr, and library composition differences were normalized using the TMM method [27,40]. Normalized expression values were converted to log2 (CPM + prior count) for principal component analysis, sample correlation, hierarchical clustering, and expression heatmaps. PCA was performed on the 3000 genes with the highest variance; Pearson correlation coefficients were used to assess between-sample expression correlation, and biweight midcorrelation (bicor) was used for network quality control [29]. Overall sample quality was assessed by jointly considering library size, alignment rate, PCA position, inter-replicate correlation, and network connectivity.

### 2.5. Differential Expression Analysis and Accession-Level Comparisons

Differential expression analysis used raw read counts as input and the edgeR quasi-likelihood generalized linear model [27,40]. DS-versus-WW expression changes were first compared separately for each of the 6 accessions. A model including accession and treatment effects (~material + treatment) was then fitted separately within the DSEN and DTOL groups to estimate the common drought effect after controlling for baseline accession-level expression differences. The conventional DEG threshold was set at FDR < 0.05 and |log2 fold change| ≥ 1, with multiple-testing correction performed using the Benjamini–Hochberg method [41].

Inference between the two groups used accessions, rather than sequencing replicates, as the independent unit. Specifically, the mean expression under WW and DS and the DS − WW response value were calculated separately for each accession, and limma v3.68.4 was used to compare expression differences between the DTOL and DSEN groups under DS conditions, as well as the interaction between group and drought response for the two groups [26]. Each group comprised 3 independent accessions. This strategy avoids mistakenly treating the 3 sequencing replicates of the same accession as independent genetic replicates, thereby reducing the significance inflation caused by pseudoreplication.

### 2.6. GO, KEGG, GSEA, and Transcription Factor Analyses

Over-representation analysis of GO and KEGG terms for DEGs was performed using clusterProfiler v4.20.0, with the background gene set restricted to genes that passed expression filtering and entered statistical testing [42]. GO terms were divided into biological process, cellular component, and molecular function categories; KEGG was used to identify metabolic and signaling pathways. The significance threshold for enrichment was set at FDR < 0.05. Transcription factor families were summarized based on PlantTFDB family mapping information, with emphasis on comparing the bHLH, ERF/AP2/RAV, MYB, NAC, bZIP, WRKY, and HD-ZIP families [43].

To reduce the information loss caused by a fixed fold-change threshold, pre-ranked gene set enrichment analysis (GSEA) was further performed on directional test statistics for all genes using fgseaMultilevel [44]. Gene set size was restricted to 10–500. FDR < 0.05 was defined as robustly significant; to display additional pathways with a consistent direction of change but relatively weaker statistical support, an exploratory threshold of FDR < 0.25 was used for the KEGG GSEA panel, with the actual adjusted *p* values retained in the figure. Exploratory results are used only to suggest trends and are not treated as definitive pathway evidence.

### 2.7. Weighted Gene Co-Expression Network Analysis

WGCNA was performed using the WGCNA v1.74 package [28]. After filterByExpr filtering, 25,099 genes were retained in the expression background set; genes were ranked by median absolute deviation (MAD) across the 36 samples, and the 10,000 most variable genes were selected for network construction. The input matrix consisted of TMM-normalized log2(CPM) expression values, with rows representing samples and columns representing genes. The goodSamplesGenes function was used to check for missing values and zero-variance genes, and hierarchical sample clustering combined with standardized network connectivity (Z.k) was used to identify potential outlier samples (samples with Z.k < −2.5 were considered for removal).

To determine the optimal soft-thresholding power, pickSoftThreshold was run with powers ranging from 1 to 30, using bicor (biweight mid-correlation) as the correlation measure to mitigate the influence of outliers, and networkType = “signed”. The minimum power at which the scale-free topology fit index (*R*^2^) first reached ≥ 0.80 while retaining reasonable mean connectivity was selected [29]. Module detection was performed using the blockwiseModules function, which constructs a signed topological overlap matrix (TOM) and recursively clusters genes. Key parameters were set as follows: networkType = “signed”, TOMType = “signed”, corType = “bicor”, minModuleSize = 40, deepSplit = 2, mergeCutHeight = 0.25, pamRespectsDendro = FALSE, and maxBlockSize = 12,000 (to accommodate the 10,000 selected genes within a single block). Highly correlated modules were subsequently merged based on module eigengene distance, and genes that could not be stably assigned to a biological module were assigned to the grey module.

### 2.8. Module–Trait Associations, Core Genes, and Sensitivity Analysis

Each module was represented by its first principal component, the module eigengene (ME). bicor was used to calculate correlations between ME and drought treatment, accession class (group), DSEN-group drought status, and DTOL-group drought status [29], with BH correction applied within each trait. The same statistical models used for differential expression were further applied to ME values: within-group DS-versus-WW comparisons controlled for accession effects, while between-group comparisons under DS and the response-interaction comparison used accession-level centroid values.

Gene module membership was represented by the correlation coefficient (kME) between gene expression and the eigengene of its assigned module, and gene significance was represented by the correlation between gene expression and the trait of interest. Core (hub) genes were prioritized by jointly considering |kME|, expression change in the target comparison, FDR, and functional annotation. GO/KEGG enrichment for key modules used the 10,000 genes entered into WGCNA as the background set. Core-gene networks were constructed based on signed adjacency relationships, and node and edge files were exported.

Sample network connectivity flagged DSEN-2_DS_R1 as a borderline low-connectivity sample. Because its alignment rate, library size, and PCA position showed no technical abnormalities and its bicor correlations with the other two replicates of the same accession were both high, this sample was retained in the primary analysis. A sensitivity analysis recalculated the module-level treatment effects for the DSEN group after excluding this sample, without altering module definitions, and compared the direction, magnitude, and ranking of the effects.

### 2.9. Physiological and Biochemical Assays

To relate transcriptomic reprogramming to physiological indicators of oxidative stress, leaf samples of uniform developmental stage were harvested in parallel with the samples used for RNA sequencing from the same six accessions under WW and DS treatments, with three independent biological replicates per accession × treatment combination. Each biological replicate consisted of pooled leaf samples from 5 to 8 individual plants, and approximately 0.5 g of fresh leaf tissue was used for each assay. Catalase (CAT) and superoxide dismutase (SOD) activities and malondialdehyde (MDA) content were determined using commercially available enzymatic/colorimetric assay kits (Solarbio Life Sciences, Beijing, China) according to the manufacturer’s instructions and are expressed as U g^−1^ fresh weight (FW) for CAT and SOD activities or nmol g^−1^ FW for MDA content. Differences between DS and WW within each accession were assessed using Student’s t-tests. In addition, two-way analysis of variance (ANOVA) with group (DSEN vs. DTOL) and treatment (WW vs. DS) as fixed factors was used to test group, treatment, and group × treatment interaction effects. All analyses were performed in SPSS 26.0 (IBM, Armonk, NY, USA), and statistical significance was set at *p* < 0.05.

### 2.10. RNA Extraction, cDNA Synthesis, and qRT-PCR Validation

To independently validate the RNA-seq-based expression patterns of prioritized WGCNA hub genes, six candidate genes from the blue module (Seita.4G007900, Seita.8G228200, Seita.4G180900) and the brown module (Seita.3G093600, Seita.5G381100, Seita.8G115400) were selected for quantitative real-time PCR (qRT-PCR) validation in the same 6 accessions under WW and DS treatments. Total RNA was extracted from the same leaf material used for library construction using TRIzol reagent (Invitrogen Thermo Fisher Scientific, Shanghai, China), following the manufacturer’s protocol; RNA integrity and concentration were verified prior to reverse transcription; all samples had A260/A280 ratios between 1.9 and 2.1, and RNA concentrations ranged from 500 to 1000 ng/µL. For each sample, 1 µg of total RNA was used for first-strand cDNA synthesis. Gene-specific primer pairs were designed for each candidate gene; primer sequences, amplicon lengths, and the reference gene used for normalization are provided in Supplementary Table S1. qRT-PCR was performed with a SYBR Green-based master mix, and relative expression was calculated using the 2^−ΔΔCt^ method. Differences between WW and DS treatments within each accession were assessed using two-tailed Student’s *t*-tests, with significance denoted as *p* < 0.05 (*), *p* < 0.01 (**), and *p* < 0.001 (**); ns indicates no significant difference.

## 3. Results

### 3.1. Transcriptome Data Quality Was Reliable, and Drought Treatment Drove the Major Expression Variation

**Figure 1:**
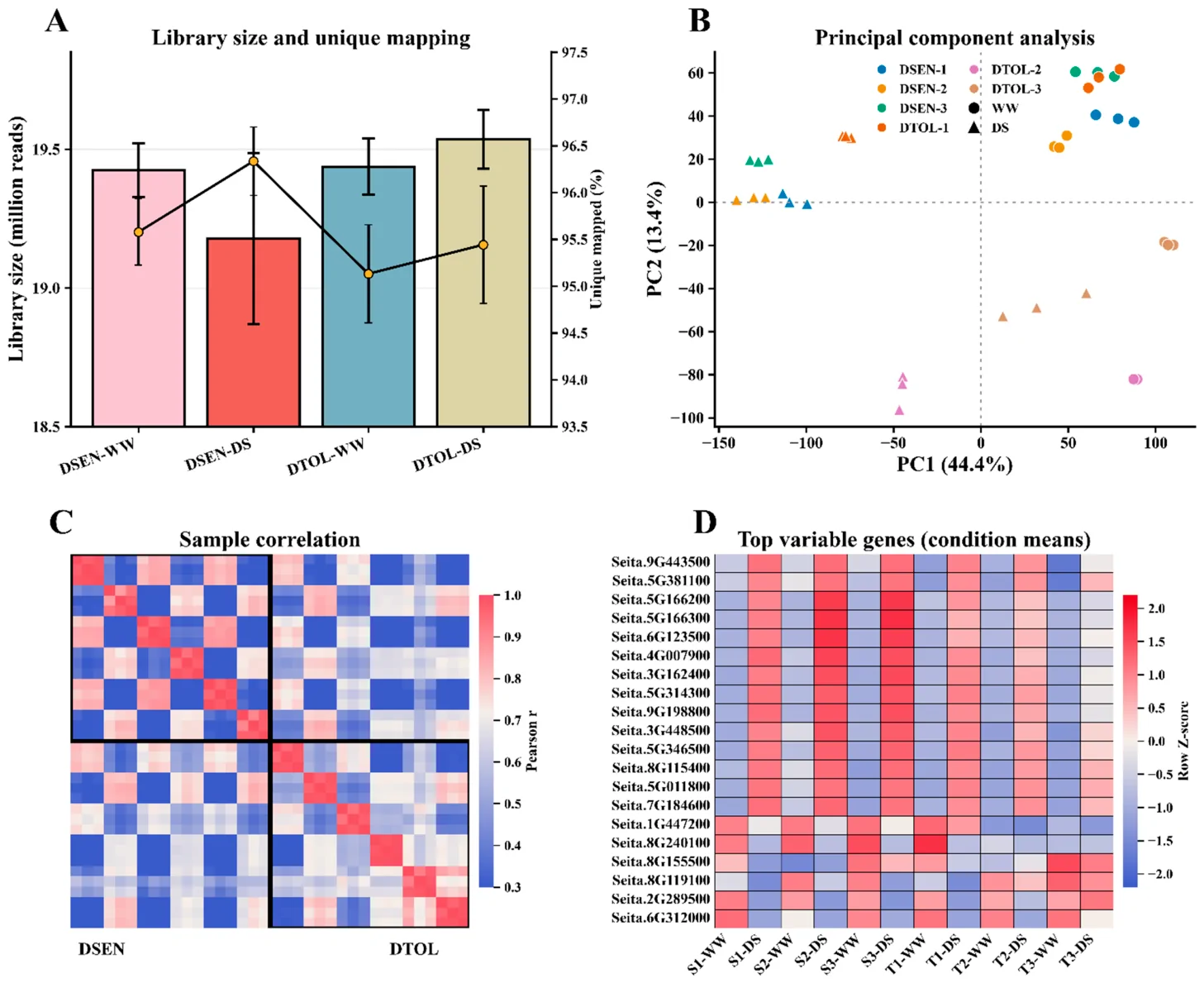
Transcriptome sequencing quality and overall expression structure of the 36 samples. (**A**) Mean library size and standard deviation for the DSEN and DTOL groups under WW and DS treatments; the line plot on the right axis shows the unique mapping rate. The left axis is displayed over the range of 18.5–19.85 million reads to visualize between-group differences without altering the raw values. (**B**) Principal component analysis based on highly variable genes. Colors indicate the 6 accessions; circles and triangles represent WW and DS treatments, respectively; PC1 and PC2 explain 44.4% and 13.4% of the expression variance, respectively. (**C**) Pearson correlation heatmap of pairwise sample expression based on highly variable genes. (**D**) Heatmap of the top 20 highly variable genes across the means of the 12 accession–treatment conditions; expression values are Z-score normalized by gene. DSEN, drought sensitive; DTOL, drought tolerant.

The 36 samples yielded an average of 43.30 million raw reads, ranging from 41.43 to 43.39 million. The average overall alignment rate was 98.67% (range 97.77–99.19%), and the average unique mapping rate was 95.62% (range 94.48–96.78%), indicating that the sequencing data could be stably aligned to the foxtail millet reference genome (Figure 1A). After filtering out lowly expressed genes, 25,099 genes were retained for downstream analysis.

PCA showed that PC1 and PC2 explained 44.4% and 13.4% of the overall variation, respectively. Most WW and DS samples showed a clear separation along PC1, while different accessions showed genetic background differences along PC2 and in their within-group positions (Figure 1B). The sample correlation heatmap showed that the three biological replicates of the same accession under the same treatment generally had high correlation with one another (Figure 1C). The heatmap of highly variable genes at the condition-mean level further showed that water treatment and accession background jointly shaped the expression structure (Figure 1D). These results support the subsequent differential expression and WGCNA analyses.

### 3.2. Drought-Sensitive Accessions Underwent More Extensive Transcriptional Perturbation, but the Two Groups Shared the Main Response Framework

**Figure 2:**
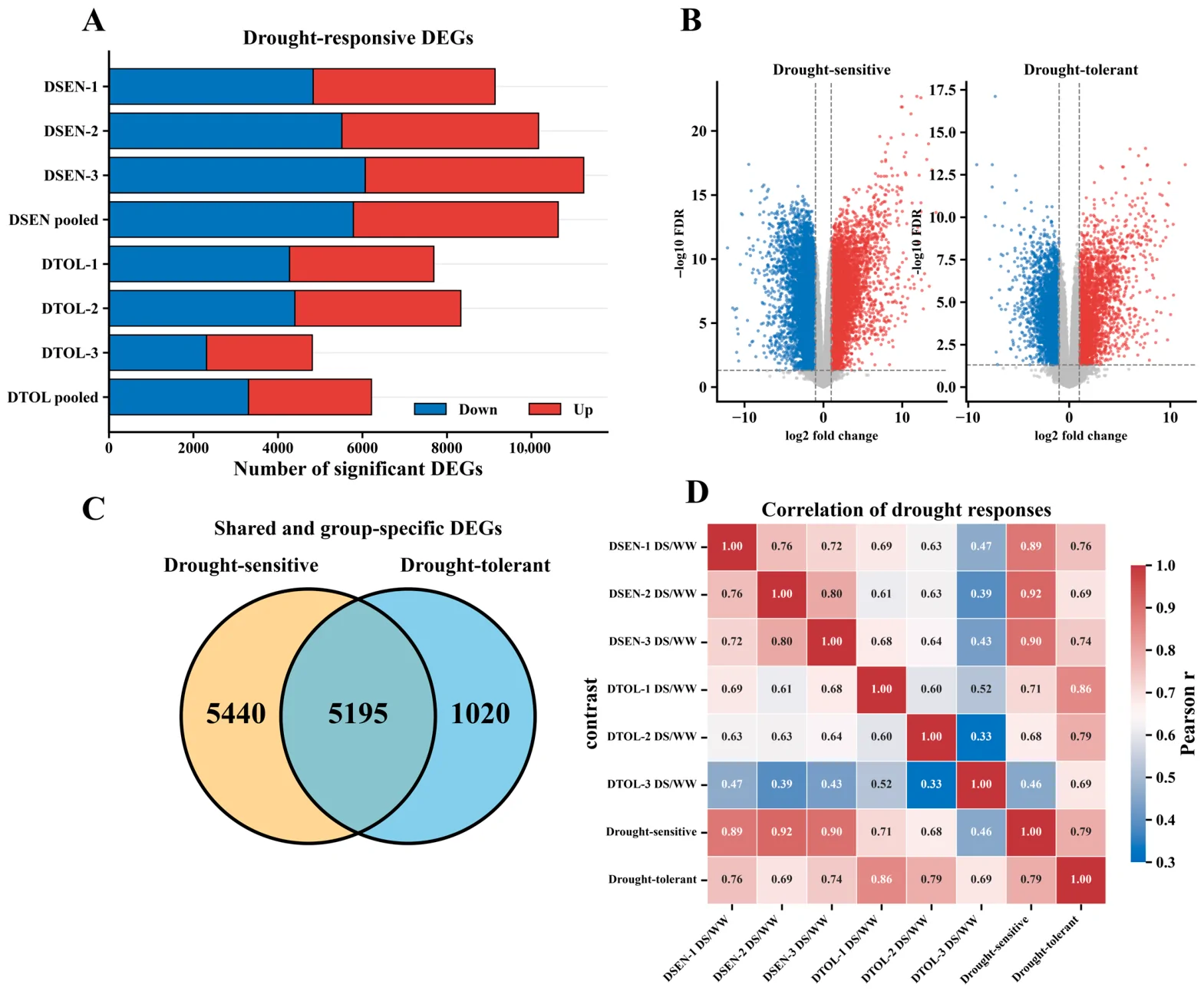
Scale of differential expression and shared drought response in drought-sensitive and drought-tolerant foxtail millet accessions. (**A**) Number of significant DEGs for the 6 individual accessions and for the pooled comparisons of the two groups. Red and blue indicate up- and down-regulated genes, respectively. (**B**) Volcano plots of the DS-versus-WW comparisons for the DSEN and DTOL groups. Dashed lines indicate the significance threshold (−log10 FDR = 1.3, corresponding to FDR < 0.05) and fold-change thresholds (log2FC = ±1). (**C**) Venn diagram of drought-responsive DEGs shared between and specific to the DSEN and DTOL groups. (**D**) Correlation heatmap of drought-response log_2_FC values among the 6 accessions and the two overall group-level responses; numbers within cells are Pearson correlation coefficients.

Under the threshold of FDR < 0.05 and |log_2_FC| ≥ 1, DSEN-1, DSEN-2, and DSEN-3 identified 9141, 10,171, and 11,238 DEGs, respectively, while DTOL-1, DTOL-2, and DTOL-3 identified 7692, 8332, and 4814 DEGs, respectively (Figure 2A). After controlling for accession effects, the DS-versus-WW comparison identified 10,635 DEGs in the DSEN group (4849 up-regulated, 5786 down-regulated) and 6215 DEGs in the DTOL group (2913 up-regulated, 3302 down-regulated) (Figure 2A). Volcano plots of the group-level comparisons visually illustrated that the DSEN group underwent more extensive significant expression changes (Figure 2B). The number of DEGs in the DSEN group was approximately 1.71-fold that of the DTOL group, and its median |log_2_FC| (1.971) was also higher than that of the DTOL group (1.745), indicating that the drought-sensitive accessions experienced more extensive and more intense expression perturbation. The two groups shared 5195 drought-responsive DEGs, with 5440 and 1020 group-specific DEGs in the DSEN and DTOL groups, respectively (Figure 2C). The Pearson correlation of the genome-wide drought response between the two groups was 0.791; the correlations between the three individual DSEN accessions and the group-level response ranged from 0.887 to 0.915, whereas those for the three DTOL accessions ranged from 0.691 to 0.856 (Figure 2D). Thus, the two groups of accessions shared the same overall direction of drought response but differed markedly in the scope and magnitude of that response.

### 3.3. Photosynthesis Was Suppressed, Accompanied by Reprogramming of Carbon Metabolism, Membrane Systems, and Defense Processes

**Figure 3:**
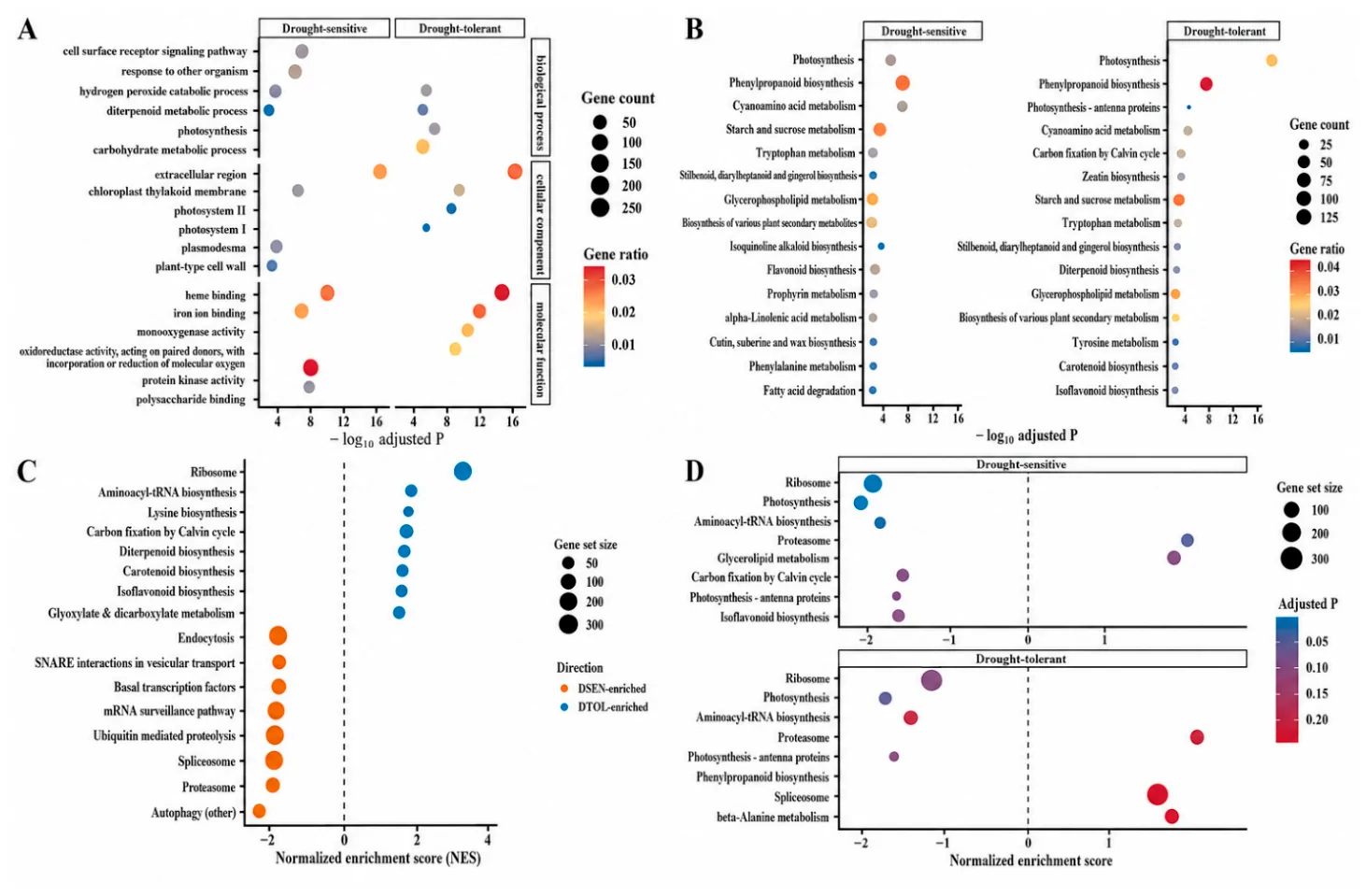
Functional enrichment and direct pathway comparison between DSEN and DTOL materials. (**A**) GO enrichment analysis of drought-responsive differentially expressed genes in the DSEN and DTOL groups. Dot size represents gene count, dot color represents gene ratio, and the x-axis represents −log10 of the Benjamini–Hochberg adjusted *p* value. (**B**) KEGG enrichment analysis of drought-responsive differentially expressed genes in the DSEN and DTOL groups. Dot size represents the number of pathway-annotated genes, and dot color represents gene ratio. (**C**) Direct KEGG-GSEA comparison between DTOL and DSEN under DS, addressing the request for a formal between-group pathway comparison rather than separate within-group analyses. Genes were ranked by the signed test statistic from the direct contrast. Positive NES values indicate enrichment toward DTOL, and negative values indicate enrichment toward DSEN. Dot size represents gene set size; the eight pathways with the strongest NES in each direction among pathways with adjusted *p* < 0.05 are shown. (**D**) Within-group KEGG-GSEA results for DS relative to WW in DSEN and DTOL. The x-axis shows NES, dot size represents gene-set size, and dot color represents adjusted *p*.

GO enrichment showed that the drought responses of both groups jointly involved photosynthesis, chloroplast thylakoid membrane, photosystem I/II, carbohydrate metabolism, cell-wall organization, hydrogen peroxide catabolism, protein kinase activity, and heme and iron ion binding (Figure 3A). Significant terms were distributed across all three ontology categories—biological process, cellular component, and molecular function—with the DTOL group showing a slightly higher number of significant terms (Figure 3C). Down-regulated genes in both groups were significantly enriched in photosynthesis- and chloroplast-related processes, suggesting systemic suppression of light capture, electron transport, and carbon assimilation. At the same time, terms related to receptor signaling, redox reactions, cell wall, and polysaccharide metabolism were reprogrammed, reflecting a reallocation of stress-defense resources concurrent with restricted investment in growth.

KEGG enrichment further showed that the DSEN group was significantly enriched for photosynthesis, phenylpropanoid biosynthesis, starch and sucrose metabolism, glycerophospholipid metabolism, flavonoid biosynthesis, alpha-linolenic acid metabolism, and cutin, suberine and wax biosynthesis; in addition to photosynthesis and phenylpropanoid metabolism, the DTOL group was also enriched for starch and sucrose metabolism, zeatin biosynthesis, tryptophan and diterpenoid metabolism, carotenoid biosynthesis and glycerophospholipid metabolism (Figure 3B). These results indicate that both groups of accessions respond to water deficit through carbon allocation, membrane lipid homeostasis, epidermal barrier formation, and secondary metabolite regulation, although the enrichment strength of individual pathways differed between the groups. To enable a direct, integrated comparison rather than two separate analyses, GO and KEGG enrichment for the DSEN and DTOL gene sets were computed with a common background and displayed jointly in the same panels (Figure 3A,B, labeled S and T, respectively), allowing shared and group-specific pathways to be compared side by side rather than interpreted in isolation.

To directly compare pathway-level differences between the two groups under drought stress, rather than inferring them indirectly from separate within-group analyses, we performed a pre-ranked KEGG gene set enrichment analysis (GSEA) using the genome-wide ranked list from the direct DTOL-versus-DSEN contrast under DS conditions (Figure 3C). This analysis revealed 39 KEGG pathways that reached Benjamini–Hochberg adjusted *p* < 0.05. Pathways positively enriched in the DTOL group (positive NES) included ribosome (NES = 3.55), aminoacyl-tRNA biosynthesis (NES = 2.07), and carbon fixation by Calvin cycle (NES = 1.86). In contrast, pathways enriched toward the DSEN group (negative NES) included autophagy-other (NES = −2.37), proteasome (NES = −1.95), spliceosome (NES = −1.92), and ubiquitin-mediated proteolysis (NES = −1.90). This direct comparison indicates that, while both groups share the same core stress-response pathways, drought-tolerant accessions maintain stronger enrichment of translation and carbon-assimilation pathways, whereas drought-sensitive accessions show heightened enrichment of protein-degradation and RNA-processing pathways under drought stress.

In addition to the direct DTOL-versus-DSEN comparison described above, we also performed within-group GSEA (DS versus WW) separately for each group to identify pathways that respond to drought within each genotype class (Figure 3D). Under the strict FDR < 0.05 threshold, both groups showed negative enrichment of ribosome and photosynthesis pathways, consistent with the shared suppression of translation and photosynthetic investment observed in the ORA. However, the DSEN group uniquely showed positive enrichment of proteasome and negative enrichment of aminoacyl-tRNA biosynthesis, whereas the DTOL group did not show these signatures at the strict threshold, suggesting that protein-homeostasis pathways are more strongly perturbed in the sensitive accessions. Using the exploratory FDR < 0.25 threshold, additional directional changes were observed in glycerolipid metabolism, the Calvin cycle, photosynthesis antenna proteins, phenylpropanoid biosynthesis, the spliceosome, and beta-alanine metabolism (Figure 3D). GSEA therefore supports an overall trend of “reduced investment in photosynthesis and translation, with enhanced protein quality control and metabolic reallocation” under drought, although the exploratory pathways still require validation with independent data.

### 3.4. Differences Between the Two Groups Mainly Involved Response Intensity, Transcription Factor Composition, and a Small Number of Marker Genes

**Figure 4:**
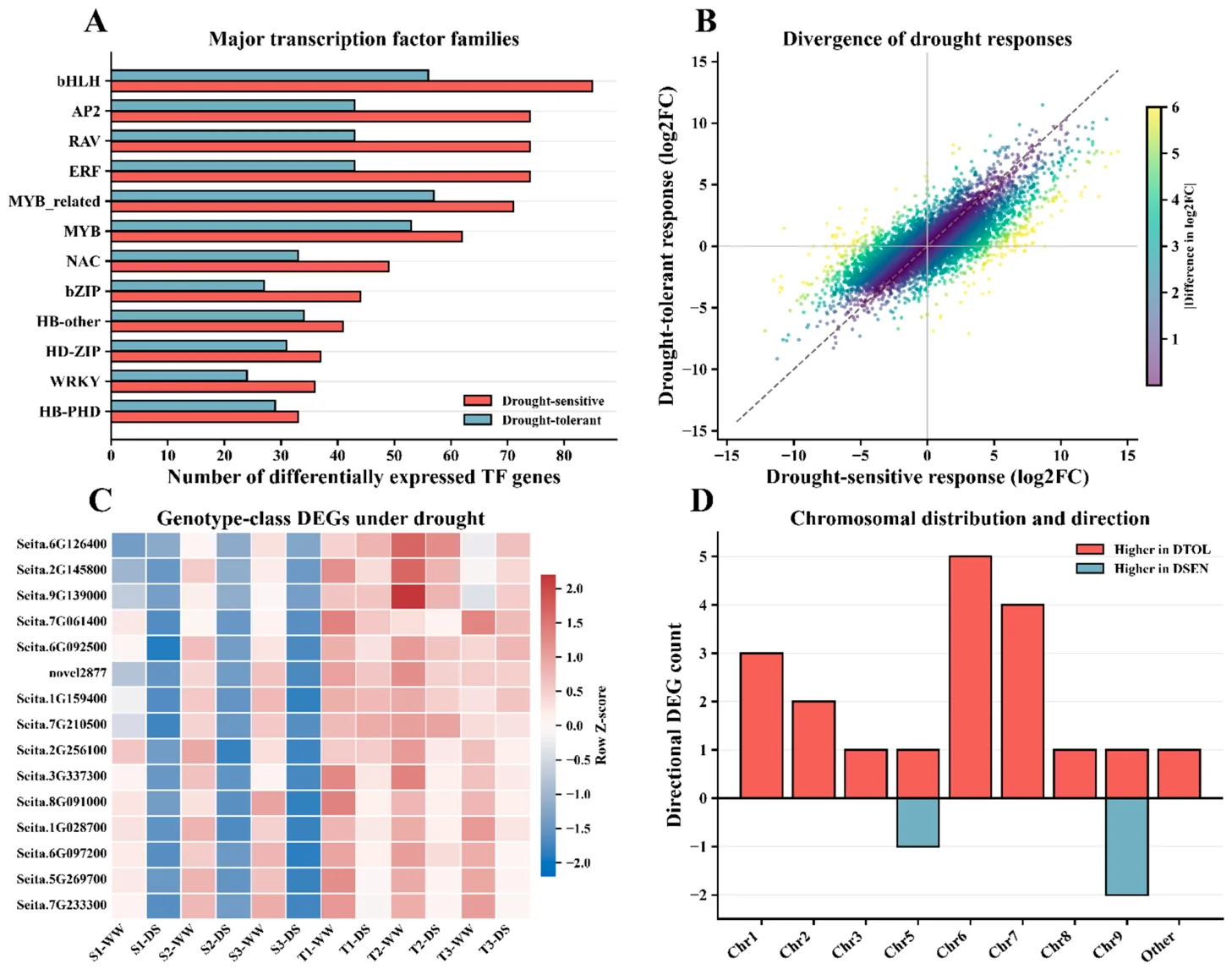
Response differences, transcription factor composition, and marker genes distinguishing the two groups under drought. (**A**) Number of differentially expressed genes in the major transcription factor families for the DSEN and DTOL groups. (**B**) Scatter plot of genome-wide drought-response log_2_FC values for the two groups; the dashed line indicates equal responses between groups, and color indicates the absolute difference in log_2_FC between groups. (**C**) Heatmap of the top 15 significant DEGs between the DTOL and DSEN groups under DS conditions, shown across the means of the 12 conditions; expression values are Z-score normalized by gene, with white lines separating cells. (**D**) Chromosomal distribution and direction of comparison for all 22 strictly significant genes (defined as genes passing FDR < 0.05 and |log_2_FC| ≥ 1 in the accession-level test between DTOL and DSEN under DS); positive red bars indicate enhanced expression in the DTOL group, and negative blue bars indicate enhanced expression in the DSEN group.

Transcription factor family analysis showed that the bHLH, ERF/AP2/RAV, MYB, NAC, bZIP, WRKY, and HD-ZIP families all underwent extensive changes in both groups, but the number of differentially expressed members in most families was higher in the DSEN group than in the DTOL group (Figure 4A). This is consistent with the larger scale of differential expression in the DSEN group and suggests that its response involves more extensive transcriptional regulatory rearrangement.

The genome-wide responses of the two groups were largely distributed along the diagonal, further indicating that the shared response predominates (Figure 4B). Accession-level statistics computed from centroid values of the three DSEN and three DTOL accessions, rather than from individual sequencing replicates (Section 2.5), showed that no genes passed the strict differential threshold between the DTOL and DSEN groups under WW conditions; under DS conditions, only 22 genes reached FDR < 0.05 and |log_2_FC| ≥ 1, of which 19 were more highly expressed in the DTOL group and three were more highly expressed in the DSEN group (Figure 4C). These genes were distributed across Chr1, Chr2, Chr3, Chr5, Chr6, Chr7, Chr8, Chr9, and unplaced scaffolds, with relatively more DTOL-up genes located on Chr6 and Chr7 (Figure 4D). No genes passed the strict threshold for the group × drought-response interaction comparison. Therefore, the tolerance-related differences should not be described as two entirely independent transcriptional programs but more likely arise from differences in a small number of marker genes, response magnitude, and module coordination within a shared network.

### 3.5. WGCNA Identified 16 Biological Modules and the Main Drought-Associated Networks

**Figure 5:**
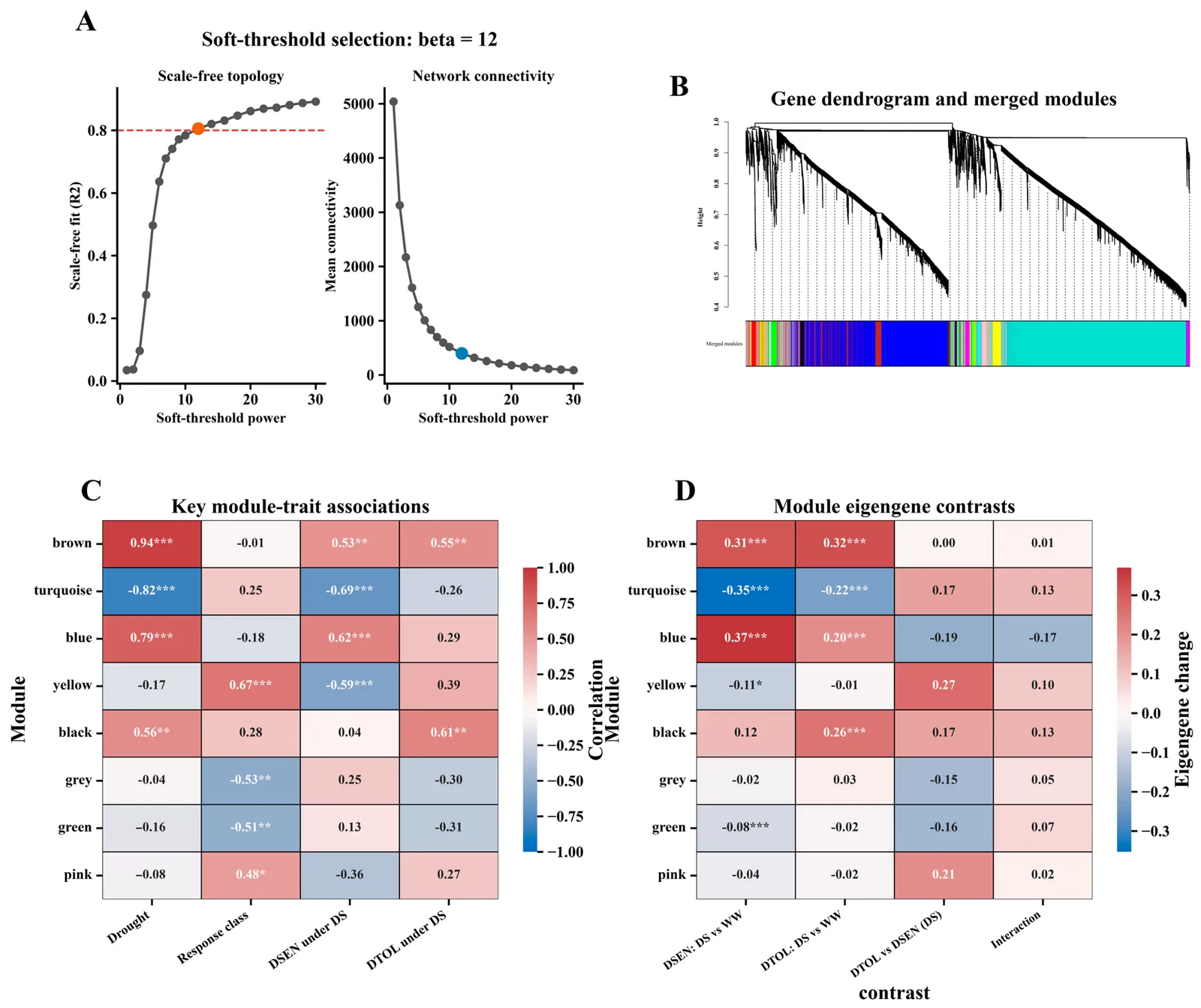
WGCNA network construction and module–trait associations. (**A**) Soft-threshold power selection. Left, signed scale-free topology fit index; right, mean connectivity; β = 12 was ultimately selected. (**B**) Gene clustering dendrogram and merged co-expression modules, with different colors representing different modules. (**C**) bicor correlation heatmap for the 8 modules most strongly associated with the experimental traits; numbers indicate correlation coefficients, and asterisks indicate BH-corrected FDR (* FDR < 0.05, ** FDR < 0.01, *** FDR < 0.001). (**D**) Heatmap of module eigengene differential tests for the same 8 modules. Columns correspond to DSEN DS vs. WW, DTOL DS vs. WW, DTOL vs. DSEN under DS, and the group × drought-response interaction. All between-group and interaction comparisons were performed using accession-level centroid values (3 accessions per group), not individual sequencing replicates, to maintain conservative statistical inference. Numbers within cells indicate effect sizes; asterisks denote BH-adjusted FDR (* FDR < 0.05, ** FDR < 0.01, *** FDR < 0.001).

A signed bicor network was constructed from the 10,000 genes with the highest MAD among the 25,099 expressed genes. At a soft-threshold power of β = 12, the scale-free topology fit index *R*^2^ reached 0.806 while retaining reasonable mean connectivity (Figure 5A). After dynamic tree cutting and module merging, 16 non-grey modules were obtained, with an additional 865 genes assigned to the grey module. The turquoise, blue, and brown modules contained 4052, 2856, and 671 genes, respectively, making them the three largest biological modules (Figure 5B).

Module–trait correlation analysis showed that the brown module had the strongest positive correlation with drought (*r* = 0.938, FDR = 5.82 × 10^−16^), the blue module was significantly positively correlated with drought (*r* = 0.786, FDR = 7.64 × 10^−8^), and the turquoise module was significantly negatively correlated with drought (*r* = −0.816, FDR = 1.13 × 10^−8^) (Figure 5C). Module eigengene differential tests further showed that, in the DSEN group, the blue module showed the most significant increase (effect = 0.370, FDR = 1.34 × 10^−15^), accompanied by a significant decrease in the turquoise module; in the DTOL group, the brown module showed the most significant increase (effect = 0.321, FDR = 8.32 × 10^−10^), the black and blue modules were also significantly increased, and the turquoise module was significantly decreased (Figure 5D). These results indicate that the two groups of accessions share the same major stress-related modules brown, blue, and turquoise but mobilize them to different degrees. The remaining 865 genes that could not be robustly assigned to any of the 16 biological modules were placed in the grey category. By construction, grey-module genes are those whose co-expression signal with other genes fell below the threshold required for stable module membership under the topological overlap and dynamic-tree-cut criteria used here; such genes are typically a mixture of comparatively low-variance transcripts, genes with idiosyncratic or noisy expression patterns not shared by other genes, and, in some cases, transcripts with less reliable quantification. Because these genes lack a coherent co-expression signal by definition, they are not amenable to the same hub-gene or functional-enrichment analyses applied to the named modules, and we therefore do not assign a specific biological interpretation to the grey module; it is treated as an unassigned background category rather than a candidate regulatory network.

### 3.6. The Blue and Brown Modules Respectively Reflect Widespread Stress Remodeling and Concentrated Signaling Regulation

**Figure 6:**
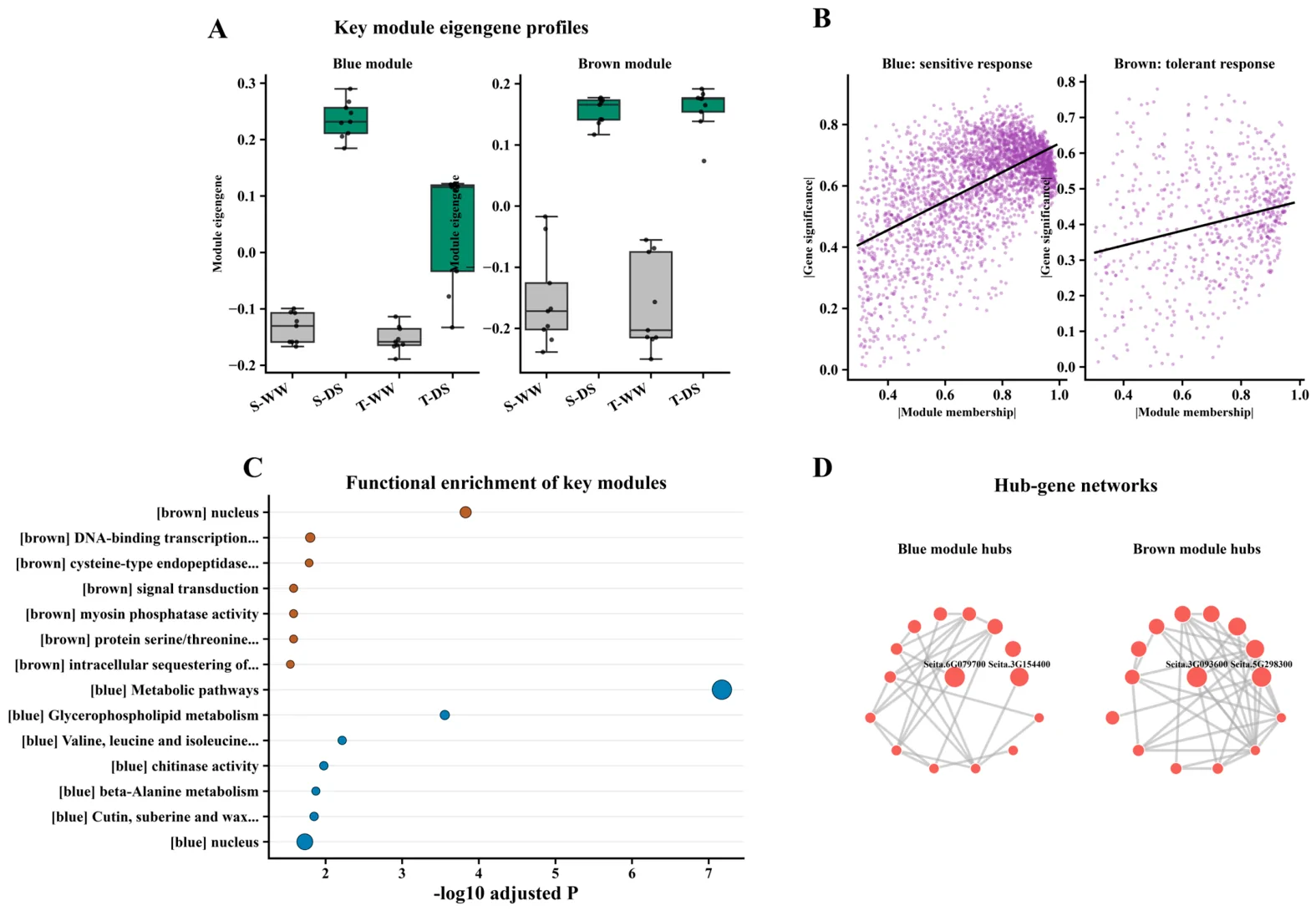
Expression, function, and core-gene networks of the key blue and brown modules. (**A**) Distribution of the blue and brown module eigengenes across drought-sensitive/drought-tolerant accessions and WW/DS treatments. (**B**) Relationship between module membership |kME| and gene significance |GS| for the target trait, with black solid lines indicating the linear trend. (**C**) Representative GO and KEGG enrichment terms for the blue and brown modules; dot size indicates the number of enriched genes, and color distinguishes modules. (**D**) High-priority core-gene networks for the blue and brown modules; node size is positively correlated with hub score, edges indicate signed adjacency strength, and the top 3 priority candidate genes for each module are labeled.

The blue module showed a marked increase under DS treatment in the DSEN group, whereas the brown module was activated in both groups of accessions and showed a highly consistent response in the DTOL group (Figure 6A). Module membership was positively correlated with gene significance, indicating that highly connected genes within a module also tended to show stronger responses in the target drought comparisons (Figure 6B).

The blue module was enriched for GO terms including chitinase activity, polysaccharide and cell-wall macromolecule catabolism, carbohydrate metabolism, endoplasmic reticulum membrane, and cis-regulatory region DNA binding; KEGG enrichment involved glycerophospholipid metabolism, branched-chain amino acid degradation, beta-alanine metabolism, cutin/suberine/wax biosynthesis, fatty acid metabolism, galactose metabolism, and secondary metabolite biosynthesis (Figure 6C). High-priority candidate genes included *Seita.4G007900* and *Seita.9G420500* (LEA proteins), *Seita.8G228200* (stachyose synthase), *Seita.4G180900* (*MAPKKK1*), *Seita.5G375800* (osmotin-like protein), *Seita.3G171800* (a C3H zinc-finger protein), and *Seita.1G307700* (an aluminum-activated malate transporter). Together, these genes point to dehydration protection, osmotic regulation, membrane lipid and cell-wall remodeling, and signaling cascades [13,15].

The brown module was significantly enriched for functions including nucleus, DNA-binding transcription factor activity, signal transduction, protein serine/threonine phosphatase activity, and intracellular sequestration of iron ions (Figure 6C). Its high-priority candidate genes included *Seita.3G093600* (calcium-dependent protein kinase 29), *Seita.9G460200* and *Seita.7G021400* (PP2C-family protein phosphatases), *Seita.5G145900* and *Seita.2G206200* (CBL-interacting protein kinases), *Seita.5G381100* (delta-1-pyrroline-5-carboxylate synthase 2), *Seita.8G115400* (dehydrin DHN1), *Seita.9G503700* and *Seita.1G253700* (HD-ZIP-related transcription factors), and *Seita.5G141600* (an AP2/ERF-related transcription factor). The co-expression network showed that these genes form a relatively tight, highly connected sub-network (Figure 6D), suggesting that drought-tolerant accessions may achieve adaptation through the concentrated coordination of calcium signaling, ABA-related dephosphorylation, proline biosynthesis, dehydration-protective proteins, and transcriptional regulation [14,15].

### 3.7. Group-Difference Modules Represent Biologically Plausible but Not Yet Rigorously Significant Candidate Networks

The yellow module was significantly correlated with DTOL-group identity; however, when the between-group test under DS conditions was performed using only the centroid values of the three DSEN and three DTOL accessions, its effect (DTOL relative to DSEN) was 0.271, with an FDR of 0.359, which did not pass multiple-testing correction. This module was significantly enriched for diterpenoid biosynthesis and contained candidate genes such as cytochrome P450s, momilactone A synthase, ent-copalyl diphosphate synthase, wall-associated receptor kinase, and a G-type lectin receptor-like kinase. The cyan module showed the largest effect size in the response-interaction comparison (0.313) but with an FDR of 0.307, likewise failing to reach strict significance; its highly connected genes included ERF, MYB, PP2C, calmodulin-binding proteins, and an E3 ubiquitin ligase. These findings prioritize diterpenoid metabolism, cell-wall sensing, ethylene/ABA/calcium signaling, and protein ubiquitination for further validation of group differences. However, at the current stage, they cannot be defined as pathways specific to the drought-tolerant group [14].

### 3.8. Network Conclusions Were Robust to the Borderline Low-Connectivity Sample

The standardized network connectivity of DSEN-2_DS_R1 fell below the preset threshold, but its sequencing and alignment metrics were normal, and its bicor correlations with the other two DS replicates of the same accession were 0.949 and 0.969, respectively. After excluding this sample, the module-level treatment effects for the DSEN group correlated with those from the complete dataset at *r* = 0.9989, with 16 of 17 modules retaining the same direction of effect, and the blue module remained the top-ranked drought-responsive module in the DSEN group. This sample therefore does not alter the main network conclusions, and the primary analysis retained all 36 samples.

### 3.9. qRT-PCR Confirms the RNA-Seq-Based Expression Patterns of Prioritized Hub Genes

qRT-PCR was performed for six hub genes prioritized from the blue (Seita.4G007900, Seita.8G228200, Seita.4G180900) and brown (Seita.3G093600, Seita.5G381100, Seita.8G115400) modules. All six genes showed significant up-regulation under DS relative to WW in the great majority of accessions (*p* < 0.05 to *p* < 0.001 for 34 of the 36 accession-by-gene combinations tested; Supplementary Figure S1A–F), matching the up-regulated direction of change identified by RNA-seq for these genes. For the blue-module genes, the magnitude of induction was consistently larger in the three DSEN accessions than in the three DTOL accessions, mirroring the broader-scale transcriptional perturbation observed by RNA-seq in the DSEN group; Seita.4G180900 additionally showed no significant change in DTOL-3 (ns), indicating some accession-level heterogeneity even within the drought-tolerant group. For the brown-module genes, strong induction was observed in both groups, consistent with the RNA-seq finding that the brown module is activated in both DSEN and DTOL accessions but most robustly in DTOL. Overall, the qRT-PCR results corroborate the direction of change reported from RNA-seq for these prioritized hub genes and support their use as candidate markers for further validation.

### 3.10. Physiological Markers Indicate Parallel Activation of Antioxidant Defenses Under Drought

Consistent with the transcriptomic evidence for reprogramming of reactive oxygen species (ROS) homeostasis, CAT and SOD activities were significantly higher under DS than under WW in all six accessions, and MDA content was significantly higher under DS in five of the six accessions (DSEN-3 being the exception; Supplementary Figure S2A–C). These results confirm that drought stress induced both antioxidant enzyme activation and lipid peroxidation at the physiological level. Two-way ANOVA with group (DSEN vs. DTOL) and treatment (WW vs. DS) as fixed factors showed that, for CAT activity, only the treatment effect was significant (*p* < 0.001), with no significant group effect or group × treatment interaction. For SOD activity, both treatment (*p* < 0.001) and group (*p* < 0.001) effects were significant, with no significant interaction; SOD activity was significantly higher in DTOL than in DSEN under both WW and DS conditions (*p* < 0.01). For MDA content, both treatment (*p* < 0.01) and group (*p* < 0.05) effects were significant, with no significant interaction. Together, these results indicate that DTOL accessions maintain higher SOD activity than DSEN accessions under both WW and DS conditions, whereas CAT responds similarly to drought in both groups. Together, these results indicate that DTOL accessions maintain higher constitutive SOD activity than DSEN accessions under both WW and DS conditions and accumulate more MDA under drought, whereas CAT responds similarly to drought in both groups.

## 4. Discussion

### 4.1. Drought-Tolerance Differences Are Better Explained by Quantitative Reprogramming of a Shared Core Network

Comparative transcriptome studies often interpret significant within one group but not the other as a group-specific response, but a difference in significance is not equivalent to a difference in response. In the present study, the two groups shared 5195 drought-responsive DEGs, and the genome-wide response correlation reached 0.791; no genes passed the strict interaction test, and only 22 genes were significant in the between-group comparison under DS conditions. These results indicate that the drought-sensitive and drought-tolerant accessions primarily engage the same drought-response backbone, with differences concentrated in response magnitude, the scope of affected genes, and module coordination. A similar pattern of a shared core response with quantitative divergence has also been reported in comparative transcriptome studies of foxtail millet [22,24,25], sorghum [33], maize [32], wheat [15,45], and halophyte species such as Puccinellia tenuiflora [46].

The DSEN group identified markedly more DEGs than the DTOL group. A larger number of DEGs should not be simplistically interpreted as reflecting stronger drought tolerance; it may instead reflect more severe perturbation of cellular homeostasis requiring more extensive compensatory transcriptional adjustment. Conversely, the smaller but more concentrated expression changes in the drought-tolerant accessions may represent pre-existing physiological preparedness, a lower degree of damage, or more efficient signaling coordination [11,15]. This interpretation is consistent with the view that drought-tolerant crops reduce global expression perturbation by maintaining membrane integrity, osmotic homeostasis, and photosynthetic recovery capacity [12,14,45,47], although this still requires validation with independent physiological indicators and additional accessions. Consistent with this interpretation, the physiological data (Supplementary Figure S2A–C) showed that drought-tolerant accessions maintain significantly higher constitutive SOD activity than sensitive accessions under both WW and DS conditions, although they also accumulated more MDA under drought, suggesting that lipid peroxidation and antioxidant capacity are not strictly inversely coupled in these accessions.

### 4.2. Photosynthetic Suppression and Resource Reallocation Form the Common Response Axis

Genes related to photosynthesis, photosystem I/II, light-harvesting antenna, and the chloroplast thylakoid were broadly down-regulated in both groups, and GSEA also showed persistent negative enrichment of photosynthesis. Drought-induced stomatal closure, reduced CO_2_ supply, and restricted electron transport can lead to excess excitation energy that further increases oxidative stress [7,14,48,49]. Reducing investment in the photosynthetic apparatus and the translational machinery is therefore a common short-term protective strategy [15,50,51]. The direct GSEA comparison between DTOL and DSEN under drought (Figure 3C) further supports this interpretation: drought-tolerant accessions maintained stronger enrichment of translation (ribosome, aminoacyl-tRNA biosynthesis) and carbon-fixation pathways, whereas sensitive accessions showed heightened enrichment of protein-degradation pathways (proteasome, ubiquitin-mediated proteolysis, autophagy). These results suggest that tolerant accessions may preserve protein synthesis and photosynthetic carbon assimilation more effectively under stress, while sensitive accessions engage more extensive protein turnover and RNA-processing adjustments. We emphasize that the additional pathways surfaced only at the exploratory GSEA threshold of FDR < 0.25 (e.g., glycerolipid metabolism, the spliceosome, and beta-alanine metabolism; Figure 3D) are reported solely to indicate directional trends and are not robustly significant; they should be treated as preliminary hypotheses requiring independent validation rather than as established components of the drought response. This study also observed negative enrichment of ribosome and aminoacyl-tRNA biosynthesis alongside positive enrichment of the proteasome, indicating a shift in the balance between protein synthesis and degradation, whereby plants may maintain protein homeostasis by reducing investment in new protein synthesis while enhancing clearance of damaged proteins [11,34,52].

Enrichment of starch and sucrose metabolism, galactose metabolism, glycerophospholipid metabolism, fatty acid metabolism, cutin/suberine/wax biosynthesis, and phenylpropanoid metabolism indicates that osmotic regulation, membrane lipid turnover, epidermal barrier formation, and antioxidant secondary metabolism jointly contribute to drought adaptation [34,53]. In particular, candidate genes such as stachyose synthase, LEA, dehydrin, osmotin-like protein, and *P5CS* are respectively associated with soluble oligosaccharide accumulation, cellular dehydration protection, osmotic regulation and proline biosynthesis [13,15,54,55]. Their high connectivity within key modules provides relatively clear priorities for subsequent qRT-PCR and functional validation.

### 4.3. The Blue and Brown Modules Reflect Two Distinct Modes of Network Organization

The blue module showed the strongest effect in the DSEN group and comprised 2856 genes involved in multiple processes, including cell-wall degradation, carbohydrate metabolism, membrane lipids, secondary metabolism, and transcriptional regulation. The size and functional breadth of this module are consistent with the large-scale differential expression observed in the DSEN group, possibly reflecting simultaneous remodeling of multiple systems under stress. Components such as the MAPK cascade, LEA proteins, osmotic protectants, and transmembrane organic acid transport may jointly participate in signal amplification, ion/organic acid balance, and cellular structural protection [15,48,56].

The brown module was smaller in size but showed the most significant and consistent positive response in the DTOL group and was enriched for transcription factors, signal transduction, and protein phosphatase activity. Nodes such as CDPK, CIPK, PP2C, HD-ZIP, and AP2/ERF can link Ca^2+^ signaling, ABA/ethylene pathways, and downstream stress-gene expression [12,17,18,57,58,59,60,61]. The brown module may therefore represent a more concentrated, hierarchically clearer regulatory unit, in which upstream Ca^2+^ and hormone signals are integrated through a phosphorylation/dephosphorylation network that subsequently drives proline biosynthesis, dehydrin accumulation, and transcriptional reprogramming [15,48]. This module represents a candidate regulatory hub in drought-tolerant accessions, although network connectivity alone cannot establish causality.

### 4.4. Diterpenoid Metabolism, Receptor Kinases, and Protein Homeostasis Are Directions Warranting Further Validation

The yellow module was enriched for diterpenoid biosynthesis and contained several P450s, momilactone-synthesis-related enzymes and cell-wall-related receptor kinases; the cyan module contained genes including ERF109, MYB4, PP2C32, calmodulin-binding protein 60B and an E3 ubiquitin ligase. These functions are closely related to hormone metabolism, cell-wall integrity sensing, calcium signaling, and protein turnover [5,62,63,64]. However, the accession-level between-group tests for both modules failed to pass FDR correction. Defining them as “drought-tolerance-specific modules” would exceed the current evidence; a more appropriate characterization is as exploratory candidate networks. If validated, these functions offer plausible mechanistic routes to tolerance: diterpenoid-pathway metabolites, such as momilactones and related phytoalexins, can strengthen cell-wall-associated chemical defense and have been linked to stress signaling in other grasses, so their coordinated induction could complement structural cell-wall reinforcement; the wall-associated and G-type lectin receptor-like kinases in the yellow module are candidate sensors of cell-wall integrity that could couple mechanical or osmotic perturbation of the wall to downstream defense and hormone signaling; and the E3 ubiquitin ligase and calmodulin-binding proteins in the cyan module point toward calcium-dependent, ubiquitin-mediated turnover of signaling components, a mechanism that could fine-tune the amplitude and duration of stress signaling by targeting negative regulators for degradation. Importantly, the direct KEGG-GSEA comparison between DTOL and DSEN under DS (Figure 3C) provides the rigorous statistical framework requested for pathway-level comparisons between the two groups, complementing the module-level analyses. The robust enrichment of ribosome, aminoacyl-tRNA biosynthesis, and Calvin-cycle pathways in DTOL, alongside proteasome, autophagy, and spliceosome pathways in DSEN, supports the conclusion that tolerance differences manifest as quantitative reprogramming of shared pathways rather than qualitative pathway switches. These hypotheses are offered to guide, rather than substitute for, the qRT-PCR, haplotype, or transgenic validation needed to establish a causal role for these modules in drought tolerance. Future work should prioritize testing the relationship between module eigengenes and drought-tolerance phenotypes in a larger population of accessions, combined with qRT-PCR, metabolite profiling, haplotype analysis, or genetic transformation to validate key nodes [11,12]. Additionally, exploring epigenetic regulation through approaches such as DNA methylation profiling [65,66] could provide deeper insights into the regulatory mechanisms underlying the quantitative differences observed between tolerant and sensitive accessions.

### 4.5. Strengths and Limitations

The main strength of this study lies in the simultaneous analysis of six independent genetic accessions and the use of accession-level centroid values in between-group comparisons, avoiding the treatment of the three sequencing replicates of the same accession as independent genotypes [26,27]. Differential expression, GSEA, and WGCNA results mutually support one another, and the outlier-sample sensitivity analysis showed that the main module-level conclusions were stable [28]. At the same time, this study has several limitations: each group contained only three genotypes, which limits the statistical power for detecting strict interaction effects [10]; group classification was based on prior drought-tolerance evaluation and would benefit from concurrent physiological phenotyping under the current sampling conditions [10,47]; the prioritized hub genes were supported by qRT-PCR validation, and the oxidative-stress phenotype was supported by physiological assays (Section 3.9 and Section 3.10), but key modules and core genes still lack independent genetics-based validation (e.g., overexpression or knockout); and exploratory GSEA results and candidate modules that did not pass FDR correction cannot be treated as definitive mechanisms [3,34]. These limitations also explain why this study emphasizes quantitative divergence within a shared network rather than making excessive claims of group-specific mechanisms.

## 5. Conclusions

Through comparative analysis of 36 foxtail millet leaf transcriptomes and WGCNA, this study revealed that drought-sensitive and drought-tolerant accessions share a common response framework under drought stress, centered on photosynthetic suppression, adjustment of carbon metabolism, membrane lipid and cell-wall remodeling, protein homeostasis, and stress signaling. Drought-sensitive accessions exhibited more extensive differential expression and strong activation of the blue module, whereas drought-tolerant accessions were characterized by the concentrated coordination of Ca^2+^/hormone signaling, protein phosphorylation/dephosphorylation, transcriptional regulation, and osmotic-protection genes within the brown module. Rigorous between-group statistics did not support the existence of entirely independent, tolerance-specific pathways but instead supported quantitative reprogramming of a shared network in response intensity and organizational mode. Genes such as *Seita.4G007900*, *Seita.9G420500*, *Seita.8G228200*, *Seita.4G180900*, *Seita.3G093600*, *Seita.9G460200*, *Seita.5G145900*, *Seita.5G381100*, and *Seita.8G115400* can serve as priority candidates for subsequent expression validation and functional studies. This study provides module- and gene-level candidate resources for molecular breeding of drought tolerance in foxtail millet and offers a reusable framework for conservative statistical inference in multi-genotype comparative transcriptome studies.

## Data Availability

The raw RNA-seq data generated in this study have been deposited in the Genome Sequence Archive (GSA) of the National Genomics Data Center, China National Center for Bioinformation/Beijing Institute of Genomics, Chinese Academy of Sciences, under BioProject accession number PRJCA074057 (https://ngdc.cncb.ac.cn/bioproject/browse/PRJCA074057 accessed on 10 September 2026). The data will be publicly available upon publication.

## Supplementary Materials

The following supporting information can be downloaded at: https://www.mdpi.com/article/10.3390/biology15191757/s1, Figure S1: qRT-PCR validation of prioritized WGCNA hub genes under WW and DS treatments in the six foxtail millet accessions. Relative expression levels for (**A**) Seita.4G007900, (**B**) Seita.8G228200, (**C**) Seita.4G180900 (blue module), and (**D**) Seita.3G093600, (**E**) Seita.5G381100, (**F**) Seita.8G115400 (brown module) are shown as mean ± SE (*n* = 3 biological replicates); expression is normalized to the reference gene (Supplementary Table S1) and scaled relative to the DSEN-1 WW sample. Asterisks indicate the significance of the DS-versus-WW comparison within each accession (Student’s *t*-test; * *p* < 0.05, ** *p* < 0.01, *** *p* < 0.001; ns, not significant). DSEN, drought-sensitive; DTOL, drought-tolerant.; Figure S2: Physiological and biochemical indicators of oxidative stress under WW and DS treatments in six foxtail millet accessions. (**A**) Catalase (CAT) activity. (**B**) Superoxide dismutase (SOD) activity. (**C**) Malondialdehyde (MDA) content. Bars represent the mean ± SE (*n* = 3 biological replicates, each pooled from 5–8 plants). Asterisks indicate the significance of the DS versus WW comparison within each accession (Student’s *t*-test; * *p* < 0.05, ** *p* < 0.01, *** *p* < 0.001; ns, not significant). DSEN, drought-sensitive; DTOL, drought-tolerant; WW, well-watered; DS, drought stress; Table S1: The primers used in this research.
