# Supplementary material for: Comparative Transcriptome and Weighted Gene Co-Expression Network Analyses Reveal a Shared Core Response and Quantitatively Divergent Regulatory Modules Between Drought-Tolerant and Drought-Sensitive Foxtail Millet
Source: Biology (Basel). 2026 Oct 2;15(19):1757. doi: 10.3390/biology15191757 (PMC13664317; doi:10.3390/biology15191757)
Supplement: Supplementary file 1 [file biology-15-01757-s001.zip › biology-4486576-supplementray.pdf]

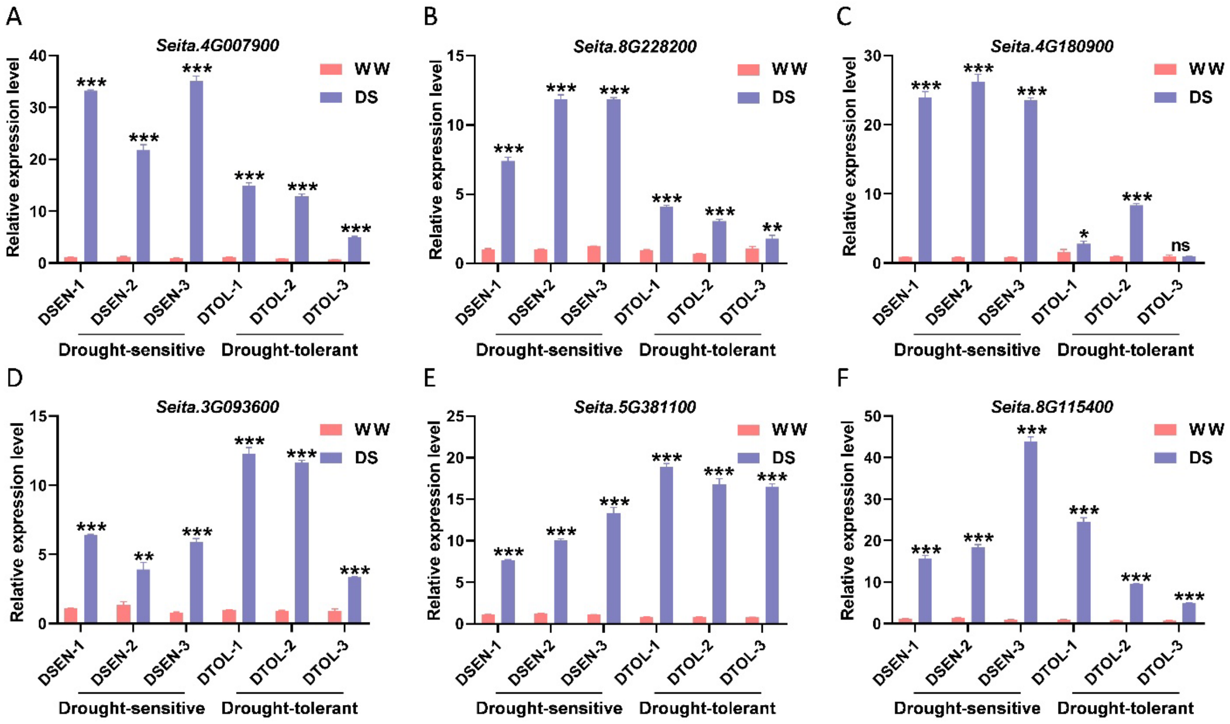

Figure S1. qRT-PCR validation of prioritized WGCNA hub genes under WW and DS treatments in the six foxtail millet accessions. Relative expression levels for (**A**) Seita.4G007900, (**B**) Seita.8G228200, (**C**) Seita.4G180900 (blue module), and (**D**) Seita.3G093600, (**E**) Seita.5G381100, (**F**) Seita.8G115400 (brown module) are shown as mean ± SE (n = 3 biological replicates); expression is normalized to the reference gene (Supplementary Table S1) and scaled relative to the DSEN-1 WW sample. Asterisks indicate the significance of the DS-versus-WW comparison within each accession (Student's t-test; * $p < 0.05$, ** $p < 0.01$, *** $p < 0.001$; ns, not significant). DSEN, drought-sensitive; DTOL, drought-tolerant.

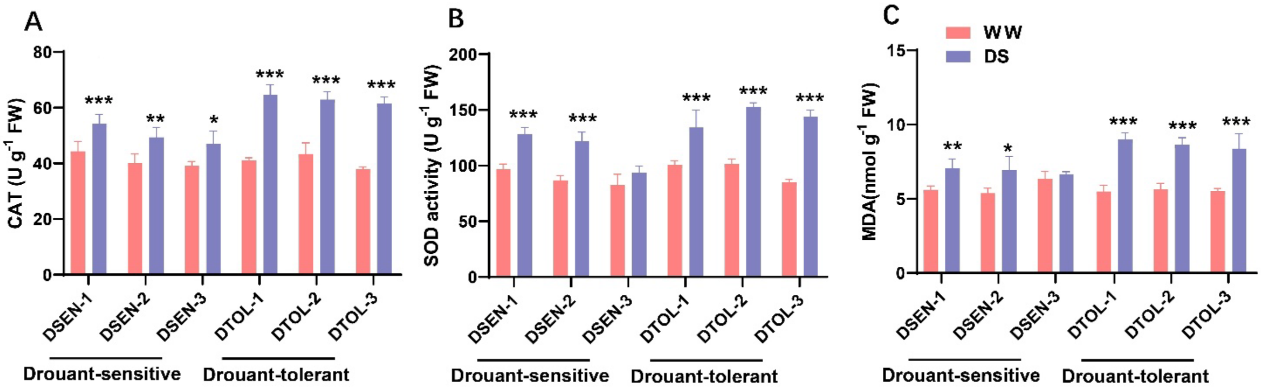

Figure S2. Physiological and biochemical indicators of oxidative stress under WW and DS treatments in six foxtail millet accessions. (**A**) Catalase (CAT) activity. (**B**) Superoxide dismutase (SOD) activity. (**C**) Malondialdehyde (MDA) content. Bars represent the mean ± SE (n = 3 biological replicates, each pooled from 5–8 plants). Asterisks indicate the significance of the DS versus WW comparison within each accession (Student's t-test; *$p < 0.05$, ** $p < 0.01$, *** $p < 0.001$; ns, not significant). DSEN, drought-sensitive; DTOL, drought-tolerant; WW, well-watered; DS, drought stress.
